# Nutrition therapy with high intensity interval training to improve prostate cancer-related fatigue in men on androgen deprivation therapy: a study protocol

**DOI:** 10.1186/s12885-016-3022-6

**Published:** 2017-01-03

**Authors:** Brenton J. Baguley, Tina L. Skinner, Michael D. Leveritt, Olivia R. L. Wright

**Affiliations:** 1 School of Human Movement and Nutrition Sciences, The University of Queensland, Brisbane, Australia; 2 Mater Research Institute, University of Queensland, Kent Street, Woolloongabba, Brisbane, QLD 4102 Australia

**Keywords:** Prostate cancer, Nutrition therapy, Exercise, Cancer related fatigue

## Abstract

**Background:**

Cancer-related fatigue is one of the most prevalent, prolonged and distressing side effects of prostate cancer treatment with androgen deprivation therapy. Preliminary evidence suggests natural therapies such as nutrition therapy and structured exercise prescription can reduce symptoms of cancer-related fatigue. Men appear to change their habitual dietary patterns after prostate cancer diagnosis, yet prostate-specific dietary guidelines provide limited support for managing adverse side effects of treatment. The exercise literature has shown high intensity interval training can improve various aspects of health that are typically impaired with androgen deprivation therapy; however exercise at this intensity is yet to be conducted in men with prostate cancer. The purpose of this study is to examine the effects of nutrition therapy beyond the current healthy eating guidelines with high intensity interval training for managing cancer-related fatigue in men with prostate cancer treated with androgen deprivation therapy.

**Methods/design:**

This is a two-arm randomized control trial of 116 men with prostate cancer and survivors treated with androgen deprivation therapy. Participants will be randomized to either the intervention group i.e. nutrition therapy and high intensity interval training, or usual care. The intervention group will receive 20 weeks of individualized nutrition therapy from an Accredited Practising Dietitian, and high intensity interval training (from weeks 12–20 of the intervention) from an Accredited Exercise Physiologist. The usual care group will maintain their standard treatment regimen over the 20 weeks. Both groups will undertake primary and secondary outcome testing at baseline, week 8, 12, and 20; testing includes questionnaires of fatigue and quality of life, objective measures of body composition, muscular strength, cardiorespiratory fitness, biomarkers for disease progression, as well as dietary analysis. The primary outcomes for this trial are measures of fatigue and quality of life.

**Discussion:**

This study is the first of its kind to determine the efficacy of nutrition therapy above the healthy eating guidelines and high intensity interval training for alleviating prostate-cancer related fatigue. If successful, nutrition therapy and high intensity interval training may be proposed as an effective therapy for managing cancer-related fatigue and improving quality of life in men during and after prostate cancer treatment.

**Trial registration:**

Australian New Zealand Clinical Trials Registry ACTRN12615000512527. Trial registered on the 22/5/2015.

## Background

Prostate cancer has become the most significant major malignancy of men, severely impacting disease-specific morbidity and mortality [1–3]. Advances in treatment of the disease, particularly through the use of Androgen Deprivation Therapy (ADT; a primary and mainstay treatment of prostate cancer), has seen prostate cancer 5-year survival rate increase to 92% [2]. Despite ADT’s efficacy in disease control, the physiological alterations resulting from ADT have profound adverse effects, including increased fatigue [4, 5], metabolic risk [6–8], cardiovascular risk [6, 9], change in body composition (increased fat mass and decreased muscle mass) [10, 11], and reduced functional capacity [12]; the amalgamation of these side effects severely reduces quality of life [13–15]. Cancer-related fatigue (CRF) is a distressing, persistent, subjective sense of physical, emotional and/or cognitive tiredness or exhaustion related to cancer or cancer treatment that is not proportional to recent activity and interferes with usual functioning [16]. CRF is the most common adverse effect of ADT, with up to 74% of men treated with ADT experiencing symptoms of CRF [4]. Incidence of CRF can be seen as early as 12 weeks after treatment initiation, and may last for longer than 12 months post-treatment in men treated with ADT [4, 5, 17]. Given the widespread use of ADT in managing prostate cancer progression, many men are living with ADT-related side effects during the course of treatment, and longer into prostate cancer survivorship. Current management strategies of CRF are predominantly through pharmacological therapy [16, 18]; yet more recently, natural therapies such as diet and exercise have demonstrated clinically significant reductions of CRF symptoms in men with prostate cancer treated with ADT [19, 20].

The benefits of aerobic exercise (performed at 65–80% age predicted maximum heart rate [APMHR] three times per week) and progressive resistance training (8–12 exercises, 2–4 sets of 8–12 repetitions performed 2–3 times per week) in isolation, or when prescribed together, are well established for improving CRF in men with prostate cancer [21–25]. Recently, there has been particular interest in the use of high intensity interval training [85–95% peak heart rate (HR_peak_) interspersed with period of active recovery] for improving CRF, body composition, and quality of life in oncological populations for managing treatment-related side effects [26–28]. In colorectal cancer survivors, 12 supervised high intensity interval training sessions (4x4 min bouts of cycling at 85–95% HR_peak_, interspersed with 3 min of active recovery at 50–70% HR_peak_) performed over 4 weeks showed greater improvements in cardiorespiratory fitness and total body mass, when compared to 12 supervised moderate intensity exercise sessions (50 min of cycling at 50-70% HR_peak_) [29]. Thus, high intensity interval training may provide an optimal exercise prescription for improving various aspects of health that are typically impaired with ADT; however exercise at this intensity is yet to be conducted in men with prostate cancer.

Combined nutrition therapy and exercise prescription has the potential to optimize management for CRF and other ADT related side effects. Aerobic exercise (55–80% APMHR) performed 2-3 times per week for 12-weeks, with healthy eating group based seminars every 2 weeks, has demonstrated clinically significant improvements in CRF and quality of life in sedentary men treated with ADT [19, 20]. In contrast individual nutrition advice to meet the United Kingdom Dietary guidelines [30] with 30 min per day of brisk walking for 24 weeks demonstrated no significant changes in CRF or quality of life compared to usual care [31]. Thus it appears CRF may be better managed with structured exercise prescription and concurrent healthy eating. Whilst Bourke and colleagues [19, 20] demonstrated significant improvements in CRF, the nutrition consults were group-based, which fails to consider individual dietary requirements and is not representative of standard dietetic practice. Thus the effects of tailored nutrition therapy beyond the healthy eating guidelines, with adjunctive structured exercise prescription on the burden of CRF remains to be elucidated.

Dietary manipulation has been identified to be an important lifestyle factor to alleviate ADT related side effects [32–34]; yet the efficacy of dietary interventions in isolation during treatment or into prostate cancer survivorship are limited for supporting the adverse side effects seen from ADT. Importantly, prostate cancer-specific dietary guidelines provide limited support and guidance for alleviating adverse treatment-related side effects, including CRF [35, 36]. In non-oncological populations, structured individualized nutrition therapy by an Accredited Practising Dietitian is recommended for adults who are overweight, obese, insulin resistant, and have altered lipid and triglyceride metabolism [37–39]; all notable adverse side effects from ADT. Yet the translation of this nutrition therapy in prostate cancer is yet to be elucidated. Recently, a Mediterranean-style diet pattern has been shown to improve metabolic and cardiovascular parameters in men at risk of prostate cancer [40]. Adherence to an anti-inflammatory properties of a Mediterranean diet have shown small reductions in hypertension (reduced systolic blood pressure; SE = – 1.44mm Hg [95% CI, -2.88 – 0.01]; and diastolic blood pressure; SE = -0.70mm Hg [95% CI, -1.34 – 0.07) [41], and risk of type 2 diabetes (RR = 0.93; 95% CI, 0.89 – 0.98) [42]; thus showing plausible metabolic and cardiovascular effects on known ADT related side effects. Yet practical application of the Mediterranean-style diet pattern to men treated with ADT for management of CRF and improving quality of life is yet to be ellucidated.

Literature to date has encompassed general healthy eating guidelines for the management of prostate cancer treated related side effects [19, 20, 31], however general guidelines do not take into account the specific dietary requirements needed to manage the ADT-related side effects. Therefore, the role of specific individualized nutrition therapy tailored to alleviate the side effects of ADT, particularly CRF, and improve quality of life warrants investigation.

This study aims to:

1. Investigate whether 12-weeks of nutrition therapy, compared to 12 weeks of usual care, can improve prostate CRF and quality of life in men treated with ADT.
2. Assess the combined benefits of 20-weeks of nutrition therapy with 8-weeks of high intensity exercise (weeks 12–20), compared to 20 weeks of usual care, on CRF and quality of life in men treated with ADT.

We hypothesized that 12-weeks of nutrition therapy, compared to 12 weeks of usual care, will improve CRF and quality of life in men with prostate cancer treated with ADT. It is further hypothesized a 20-week nutrition therapy intervention with 8 weeks of high intensity interval training, compared to 20 weeks of usual care, will improve CRF and quality of life in men with prostate cancer treated with ADT. Secondary measures of body composition, functional capacity, metabolic syndrome and biomarkers such as prostate specific antigen, insulin like growth factor [43]-1, IGF-2, IGF binding protein-3, interleukin [IL]-6, and IL-8), will be measured and analyzed between and within groups. We hypothesis nutrition therapy alone, and with high intensity exercise will improve body composition, functional capacity, and biomarkers of metabolic syndrome and prostate cancer progression.

## Methods/design

**Fig. 1:**
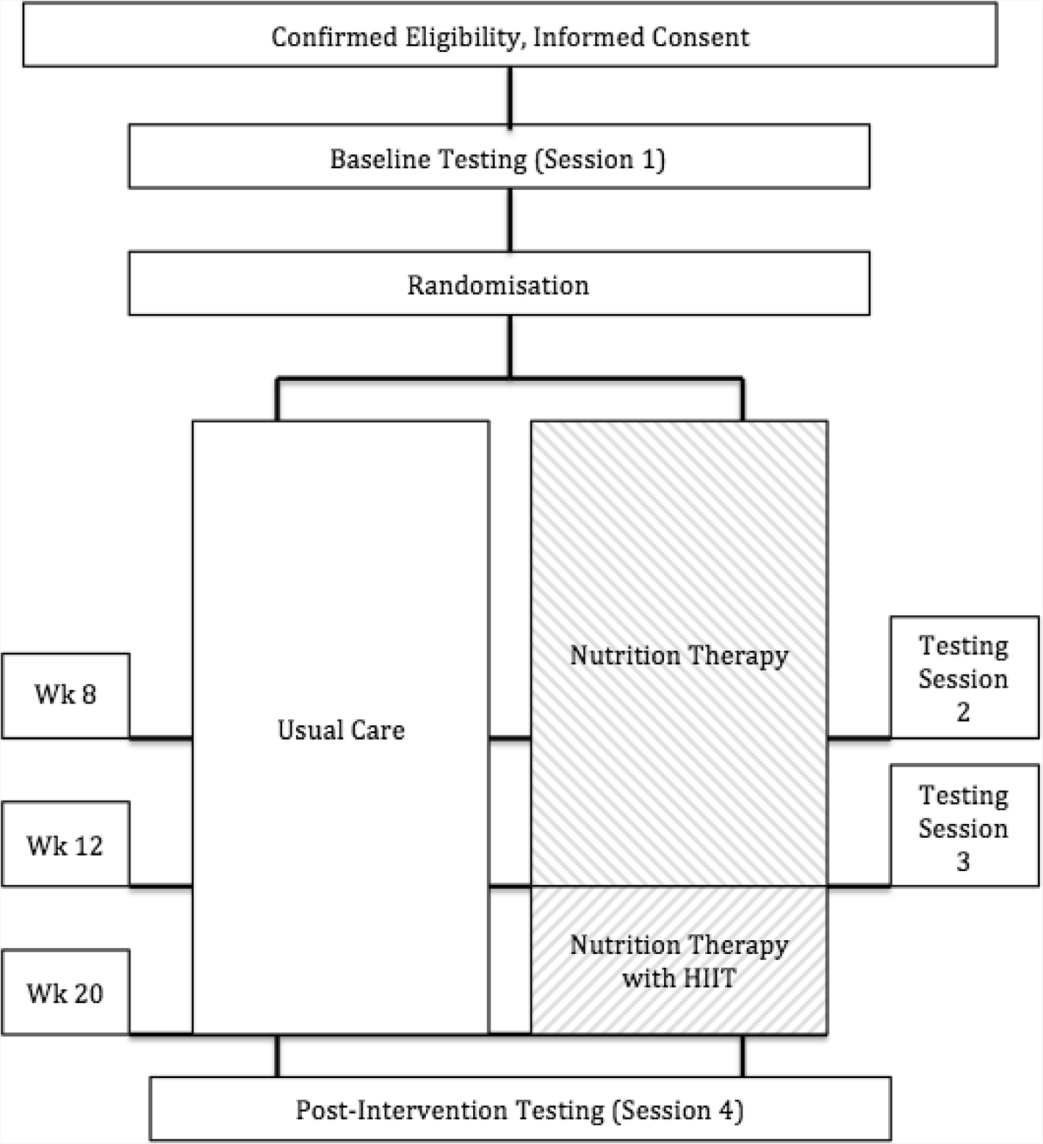
CONSORT diagram and schematic representation of the study. Legend: Wk = week, HIIT = high intensity interval training

The study is a two-arm randomized controlled trial design conducted at The University of Queensland School of Human Movement and Nutrition Sciences. All primary and secondary outcomes will be measured at baseline, week 8, 12, and 20. A total of 116 men with prostate cancer treated with ADT (>3 months) will be randomized to either a 20-week nutrition therapy with 8 weeks of high intensity exercise (performed from weeks 12 to 20) or a control group of usual care. A person independent to the study will conceal participant allocation, after baseline testing using a random number generator into either group, with equal probability. The study will be guided by the CONSORT statement [44] (Fig. 1).

### Participant eligibility

To be eligible to participate in this study, men must meet the following inclusion criteria: (a) aged ≥18 years, (b) non-smoker, or have quit smoking for ≥3 months, (c) a diagnosis of prostate cancer, and have been undertaking ADT for ≥3 months, (d) body mass index of 18.5-34.9 kg/m^2^ (i.e. normal weight, overweight or obese class I). Exclusion criteria include: (a) taking any supplements other than a single multivitamin, unless stated for medical purposes, (b) have any musculoskeletal, neurological, respiratory, metabolic or cardiovascular conditions that may prevent safe completion of the exercise demands of the study, as determined by a Urologist, (c) current infection, (d) bone metastases, (e) currently undertaking high intensity exercise i.e. ≥90% heart rate max (HR_max_) or ≥7 rating of perceived exertion (RPE; Borg’s rating of perceived exertion scale, category scale 0-10).

### Recruitment and informed consent

Participants will be recruited from the Mater Adults Hospital, Brisbane, Australia; The University of Queensland, Australia; and the Cancer Council Queensland, Australia. Participants will also be recruited by way of media releases, presentations and advertisements in newsletters, newspapers, and noticeboards at The University of Queensland, Cancer Council Queensland, and the Prostate Cancer Foundation of Australia support groups. All participants will be complete a brief telephone screening questionnaire to verify eligibility. Eligible participants will be asked to seek guidance from their Urologist regarding participation in the study, and sign the consent form prior to participating in the study. Approval of the trial protocol was obtained from the Mater Research Ethics Committee (HREC/15/MHS/38) and The University of Queensland Human Research Ethics Committee (2015001245) prior to recruitment.

### Randomisation

After baseline testing, participants will be randomly allocated to either the nutrition therapy with high intensity exercise or the standard care group using a 1:1 ratio. The randomisation process will be conducted by a computerized system using a random number generator, with equal probability, by a person external to the study.

### Measurements

All measurements for primary and secondary outcomes will take place at baseline, week 8, week 12, and week 20 (see Table 1). A familiarization trial including the functional capacity and peak aerobic power (V̇O_2peak_) tests will take place at least 7 days prior to baseline testing. Participants will be asked to refrain from vigorous exercise, caffeine, alcohol, food and beverages (except for water) for 12 h before all testing sessions.

**Table 1:** Data collection schedule

Assessments	Time (weeks)				
	Baseline^a^		Week 8	Week 12	Week 20
Screening					
Participant eligibility	✓	Randomization			
Adult Pre-exercise Screening System	✓				
Medical history form and medications	✓				
Primary outcomes					
Function Assessment of Cancer Therapy-Fatigue	✓	Randomization	✓	✓	✓
European Organization of Research and Treatment of Cancer QLQ-C30*	✓		✓	✓	✓
European Organization of Research and Treatment of Cancer QLQ-PR25^□^	✓		✓	✓	✓
The Medical Outcomes Study 36-Item Short-Form 36	✓		✓	✓	✓
Second Outcomes					
Anthropometric physique traits	✓	Randomization	✓	✓	✓
Body composition^b^	✓		✓	✓	✓
Isometric strength	✓		✓	✓	✓
Chest and leg one repetition maximum test	✓		✓	✓	✓
Sit to stand test	✓		✓	✓	✓
Six-meter walk test	✓		✓	✓	✓
400-meter walk test	✓		✓	✓	✓
Cardiorespiratory fitness^c^	✓		✓	✓	✓
Blood collection	✓		✓	✓	✓
Wollongong Dietary Inventory	✓		✓	✓	✓
24-h diet recall^d^	✓		✓	✓	✓
Depression Anxiety Stress Scale	✓		✓	✓	✓
Insomnia Severity Index	✓		✓	✓	✓
Physical Activity Enjoyment Scale	✓		✓	✓	✓
Godin Leisure-Time Physical Activity Questionnaire	✓		✓	✓	✓
Accelerometer^e,f^	✓		✓	✓	✓

^a^Nutrition and exercise intervention commences Week 0 after randomisation

^b^Dual energy X-ray absorptiometry

^c^V̇O_2peak_ test

^d^Conducted every 2 weeks

^e^Actigraph GT3X+

^f^to be worn for 7 consecutive days

*Cancer quality of life questionnaire for people with cancer

^□^Cancer quality of life questionnaire for people with prostate cancer

### Primary outcomes

#### Fatigue

Fatigue will be measured using The Functional Assessment of Cancer Therapy: Fatigue (FACT-F) questionnaire, which reports weekly fatigue on a scale of 0–52, with higher values indicating less fatigue [45]. The FACT-F has shown good reliability and validity (Cronbach’s α: 0.93-0.94) for predicting clinically significant treatment outcomes of CRF [46].

#### Cancer-specific quality of life

The European Organization for Research and Treatment of Cancer (EORTC) QLQ-C30 is a 30 item cancer-specific instrument including the following domains; physical, role, emotional, social, and cognitive, global quality of life, fatigue, pain, and emesis. The EORTC QLQ-C30 has shown a high reliability (Cronbach’s α; 0.63-.80) in cancer patients [47].

#### Prostate cancer-specific quality of life

The EORTC QLQ–PR25 is a 25 question prostate cancer-specific instrument including domains; urinary, bowel, use of incontinence aids, treatment-related symptoms, sexual activity and sexual function [48]. The EORTC QLQ – PR25 questionnaire has shown good internal reliability (Cronbach’s α; 0.70-0.86) for prostate specific treatment-related side effects [49].

#### General health and well-being

The Medical Outcomes Study 36-Item Short-Form 36 Health Survey (SF-36) is scored by eight scales encompassing physical and mental measures [50]. The SF-36 questionnaire has widely been used to measure general health and well-being, with good construct validity (Cronbach’s α; > 0.85) in cancer patients [51].

### Secondary outcomes

#### Physique traits and body composition

Height, body mass, and waist circumferences will be measured according to the International Society for the Advancement of Kinathropometry (ISAK) procedures [52].

Body composition (fat mass, lean mass, body fat percentage and bone mineral density) will be assessed using dual energy X-ray absorptiometry (DXA; Hologic Discovery A, Waltham, MA, USA).

#### Muscular Strength and Power

Isometric strength of dominate and non-dominate handgrip will be assessed using a spring-loaded grip dynamometer (TTM, Tokyo, Japan) to estimate physical performance [53], and muscular strength [54]. Participants will perform the test three times on each hand with the best result used for analysis. Muscular strength of the chest and legs will be measured using the one repetition maximum (1RM) chest and leg press methods, respectively [55]. The sit to stand test will be used to assess functional leg power [12]. Participants will perform the test three times with the best result used for analysis.

#### Exercise capacity and cardiorespiratory fitness

The six-meter walk test will involve participants walking a marked 10-m distance as quickly as safely possible, with performance timed over the middle 6-m distance to minimize the influence of acceleration and deceleration [12]. The 400-m walk test will be used to estimate exercise capacity [12]; participants will be required to walk 10 laps out and back over a 20-m course (400 m total) as fast as safely possible.

Cardiorespiratory fitness will be assessed using a V̇O_2peak_ test. The test will involve a modified ramp protocol described by Wasserman et al [56] on a cycle ergometer. Participants will begin with 3 min of rest for respiratory normalization, followed by 4 min of warm-up at a resistance of 50 Watt. The electrical resistance provided by the cycle ergometer increases incrementally by 20-30 W.min^-1^. Participants will cycle at a cadence between 60 and 70 revolutions per minute throughout the test. Heart rate will be continuously recorded throughout the exercise using a heart rate monitor (Polar FT1; Polar, Kempele, Finland) and blood pressure (Durashock Sphygmomanometer; Welch Allyn, New York, USA) will be recorded every 2 min throughout the test. At the conclusion of each minute participants will indicate their rating of perceived exertion (RPE) on the Borg 6-20 scale [57]. The test will be terminated when the participant reaches volitional fatigue or at the discretion of the researchers with consideration for exercise testing termination criteria as outlined by the American Association of Cardiovascular and Pulmonary Rehabilitation [58]. The gas analyzers and ventilometer will be calibrated prior to and verified after each test. Sampled expired air will be measured every 15 s using a turbine ventilometer (Morgan, Model 096, Kent, England). The ramp V̇O_2peak_ protocol has good validity in comparison to the standard Bruce protocol [59]. V̇O_2peak_ will be recorded as the highest V̇O_2_ reading averaged over two consecutive readings.

#### Blood collection and analysis

A trained phlebotomist will extract, treat and subsequently store the blood at approximately -80°C until later analysis. Analysis of blood samples through commercial ELISA kits (Thermo Fisher Scientific Australia Pty Ltd., Victoria, Australia; Randox Laboratories Ltd., West Virginia, USA; R&D Systems Inc., Minneapolis, USA) will be used for PSA, IGF-1, IGF-2, IGFBP3, IL-6, IL-8, Hepcidin, total cholesterol, and triglycerides analysis.

#### Dietary intake

Participants will complete the Wollongong Dietary Inventory [60] (a comprehensive dietary history of intake over the past month) with cross-checking quantification from an Accredited Practising Dietitian. Narrative approaches to diet histories have been shown to provide good reproducibility and reliability compared to food records [61]. Food models and pictures of food portions (Great Ideas in Nutrition, Coolangatta, Australia) will be utilized to improve the accuracy of food intake estimates [62].

### Other measures

#### Psychosocial: Depression Anxiety Stress Scale (DASS)

The DASS is a 42-item self-report instrument design to measure the three related negative emotional states of depression, anxiety, and stress [63], and has been validated for measuring emotional states of depression (Cronbach’s α: 0.94), anxiety (Cronbach’s α: 0.88) and stress (Cronbach’s α: 0.93) in clinical populations [64].

#### Insomnia Severity Index (ISI)

The ISI questions relate to subjective qualities of the respondents sleep, including satisfaction with sleep patterns, the degree to which insomnia interferes with daily functioning, and how the respondent feels their insomnia is noticeable to others [65]. The ISI has shown excellent internal consistency (Cronbach’s α: 0.90) for detecting symptoms of insomnia.

#### Physical activity enjoyment scale (PACES)

The physical activity enjoyment scale (PACES) will be used to assess participant enjoyment of the high intensity interval exercise PACES consists of 17 subscales that each relate to an aspect of enjoyment; along a 7-point continuum for each subscale, participants are asked to provide a rating to reflect their agreement with one of two bi-polar statements (e.g. ‘I enjoy it’ – ‘I hate it’) [66]. The PACES questionnaire has shown high internal consistency (Cronbach’s α: 0.90) in measures of exercise enjoyment [67].

#### Godin leisure-time physical activity questionnaire

The Godin leisure-time physical activity questionnaire requires participants to recall during a typical 7-day week the frequency and duration of exercise completed at three separate intensities: mild, moderate, and strenuous intensity [68]. The Godin leisure-time physical activity questionnaire has shown high agreement and validation (agreement: 70.8%) with minutes spent physically active, in comparison to an accelerometer in breast cancer survivors [69].

#### Accelerometer

Participants will be asked to maintain their current level of physical activity outside of the testing sessions for the duration of the study. Physical activity will be objectively assessed using the Actigraph GT3X+ accelerometer (Actigraph, Pensacola, Florida), a small, waist-worn, non-invasive device. Few studies have been published on the validity of the GT3X+ version of the Actigraph accelerometer specifically, however previous versions of the Actigraph accelerometer (CSA and GT1M) have demonstrated waist-worn validity in treadmill walking and running compared with indirect calorimetry (*r* = 0.56, *p* < 0.001 and *r* = 0.53, *p* < 0.05, respectively) in adults [70, 71].

### Interventions

#### Nutrition therapy with high intensity exercise

**Fig. 2:**
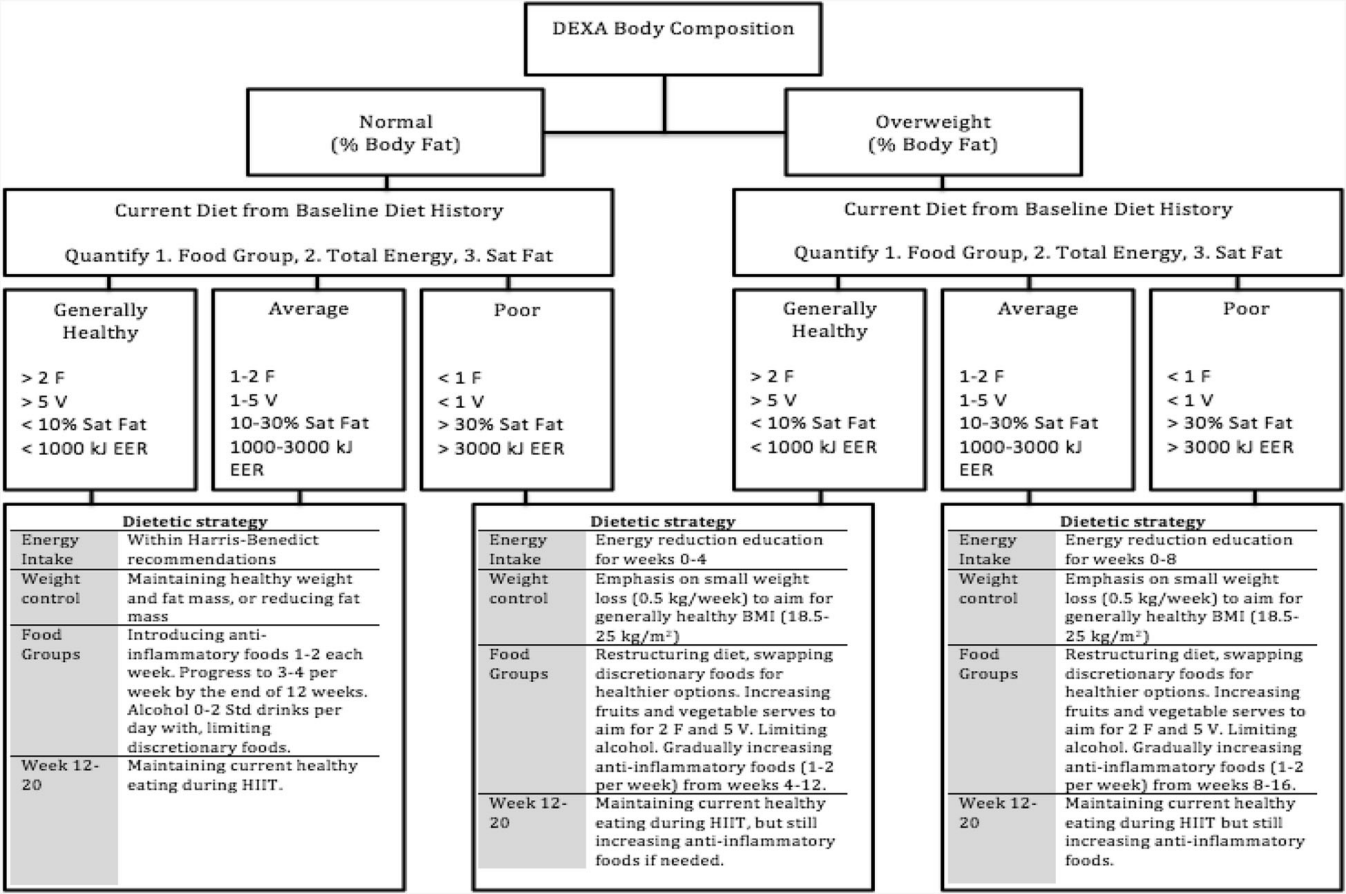
Nutrition therapy assessment schematic representation. Legend: DEXA = Dual-energy X-ray absorptiometry, F = fruit, V = vegetable, Sat fat = saturated fat, EER = estimated energy requirement, HIIT = high intensity interval training, Std = standard drink, BMI = body mass index

Detailed diet histories will be conducted at baseline week 8, 12 and 20 using the Wollongong Dietary Inventory [60], and 24 h diet recalls every two weeks during the intervention. Each consult will take approximately 30-45 min and involves an Accredited Practising Dietitian asking detailed questions about the foods consumed and the amount and frequency of consumption. The 20-week diet emphasizes a total energy intake decrement using the Harris-Benedict predicted energy requirements [72]; with a dietary composition of 45-65% carbohydrate, 20-35% fat, saturated fat <10% total energy intake, and 15-25% protein sources. Dietary advice will be tailored according to current body composition classification as ‘normal, overweight or obese stage I’ (see Fig. 2). A dietary energy reduction of 2000-4000 kJ/day will be emphasized at baseline consultation if body composition and diet intake are classified average or poor according to Fig. 2: Nutrition therapy schematic representation. The diet intervention will encompass a Mediterranean-style diet containing cruciferous vegetables (broccoli, bok choy, cauliflower) [73], tomato and tomato-paste containing products (lycopene) [74, 75], fruits, calcium and vitamin D containing foods, polyunsaturated, monounsaturated, omega 3 and 6 fatty acids (<10–15% total energy intake) [76], and a reduced consumption of red meat (<2 a week) [77, 78]; as seen in Table 2: Nutrition recommendations. Each individual nutritional consultation, will aim to make gradual changes to the participants’ diets to best achieve a modified Mediterranean-style dietary pattern. Standard dietetics practice will be used to assess and providing guidance on managing the nutritional impact symptoms from treatment (including broader intolerances/allergies).

**Table 2:** Nutrition recommendations

Food/Nutrient	Serving summary	Recommendation
Total Energy Intake	As specified by your Accredited Practising Dietitian, using the Harris-Benedict equation.	Within requirements
Saturated fat	1 serve = 1 tablespoon of butter, 1 sweet biscuit. Excess fat on meat, full-cream dairy, take-away foods, cakes, bakery items, processed meats, vegetable oil.	<10% total energy
Refined Carbohydrates	Serving size = 1 (40g) Donut, 30g potato chips, 12 hot chips, 1 can of soft drink (375ml), ½ small chocolate bar (25g)	Limit consumption
Whole Grains	1 serve = 1 slice of multigrain bread, ½ cup oats,	4-6 serves per day
Fruits and Vegetables	One serving = 1 medium piece (apple, banana, orange, pear), 2 small pieces (e.g. apricot, kiwifruit, plums), ½ cup of tomato, ½ cup or carrot, ½ cup green beans, ½ cup of pumpkin, 100g mixed frozen vegetables	2 fruits and 5 vegetables per day
Fibre	Aim for 30g/day through your grains, vegetables, and fruit intake	30g/day through grains, fruits and vegetable intake
Lentils/Beans	1 cup per day of cooked or canned legumes/beans such as lentil, chick peas or split peas	1 cup per day
Lean Meats	Lean meats 3-4 times per week. Serving size of 65g include beef, lamb, veal, port, and 80g for chicken, turkey, duck, and 2 large eggs	Lean meats 3-4 times per week
Meats with carcinogenic properties	Red charred meat, visible from over cooking or burning the mean. Processed meats (i.e. salami, sausage)	Reduce or eliminate
Oily Fish	100g per serve of fresh salmon, tuna, trout	2-3 times per week
Dairy	1 cup of fresh, reduced fat milk, or soy milk	2-3 serves per day
Nuts and Seeds	30g (handful) of almonds, cashew nuts, walnuts, macadamia nuts, brazil nuts, pumpkin seed, sunflower seeds	30g per day
Alcohol and discretionary foods	1 standard drink of alcohol = 375ml mid strength beer, 1 glass of wine, 30ml spirit. Discretionary foods = biscuits	0-2 standard serves of alcohol, and reduce or eliminate discretionary foods.

Each participant in the intervention group will be provided with a nutrition pamphlet for supportive education during the study to represent standard dietetic practice. The nutrition pamphlet will be provided at the first dietetic consult and is designed to facilitate the dietetic consultation to help maintain a Mediterranean-style diet pattern. The pamphlet consists of background information about nutrition requirements, optimal nutrition during prostate cancer based on current evidence (kilojoule/cal, fatty acids, carbohydrate, meats and protein, fruits and vegetables, cruciferous vegetables, lycopene, soy isoflavones, vitamin C and calcium, alcohol and discretionary foods) and exercise during prostate cancer treatment. The pamphlet further consists of Decision Balance Tools to help change dietary patterns if needed throughout the dietetic consults by identifying possible barriers to adopting the nutrition therapy. These form part of standard dietetics practice involving motivational interviewing.

Baseline body composition scans (DXA) will classify participants as normal, overweight, or obese state I, according to age specific normative Z values for percentage of body mass index and fat mass [79]. The current diet will be quantified into food groups, total energy intake, and saturated fat intake. From this the diet will be classified as ‘generally healthy’, ‘average’ or ‘poor’ compared to the Australian Dietary Guidelines [80] (as seen in Fig. 2: Nutrition therapy schematic representation). To improve adherence, the nutrition therapy will not be prescriptive, and instead utilize individualized food-based goals with the aim to progressively increase the intake of plant-based foods over the duration of the intervention to meet a modified Mediterranean-style diet pattern.

From weeks 12–20 participants will be required to visit The University of Queensland’s School of Human Movement and Nutrition Sciences three times per week to complete high intensity interval training sessions. Prior to exercising, participants’ heart rate and blood pressure will be measured for contraindications to commence exercising as outlined by the American College of Sports Medicine [81]. The high intensity exercise sessions will commence with 10 min of warm up at 50–70% HR_peak_ before completing 4 x 4 min bouts of cycling at 85–95% HR_peak._ Each 4 min interval will be interspersed with a 3 min period of active recovery at 50–70% HR_peak,_ totaling 38 min for the session. The high intensity exercise sessions will be conducted on an air- and magnetically-braked cycle ergometer (Wattbike Ltd., Nottingham, England). Participants will continue to receive the same dietetic intervention outlined above from weeks 12–20.

#### Usual Care

Participants randomized to usual care will continue their usual medical care during this period. Participants in the usual care group will be monitored for 20 weeks and perform identical primary and secondary outcome measures at the same time points as outlined above for the intervention group.

### Sample Size Calculation

Sample size calculations were complete using Vanderbilt Power and Sample Size software (Vanderbilt University, TN). Combined diet and exercise interventions with CRF as an outcome are relatively limited. A diet and exercise intervention in men treated with ADT showed prostate cancer-specific CRF (as measured by FACT-F) scores improved by 3.7 – 14.2 (CI, 95%; adjusted mean 8.9 points) in the intervention group compared to the control group [20]. Assuming FACT-F scores for this group were normally distributed with a SD of 8.9, a true difference between the experimental and control arms of 5.2, and a power of 0.8, we would need 47 experimental and control participants to be able to reject the null hypothesis. The type 1 error probability associated with this test of the null hypothesis is 0.05. With an anticipated 20% attrition rate based on previous research from our laboratory, we would need 58 experimental and control group participants to be able to reject the null hypothesis.

### Statistical Analysis

Data will be analyzed using the SPSS statistical software package (version 20.0, SPSS, Inc., Chicago, IL). Normality of the distribution for all outcome measures will be assessed using the Kolmogorov Smirnov test. Analyses will include standard descriptive statistics, t tests, correlation, regression and two-way repeated measures ANOVA or the comparable non-parametric test as necessary to examine differences between and within groups, at baseline and weeks 8, 12, and 20.

### Data Collection, Management and Monitoring

The principle investigator and trained research assistants will collect anthropometric, diet, exercise and lifestyle data from the study participants. Inter- and intra-tester reliability will be determined for data collecting investigators and research assistants for all outcome measures as appropriate. Computer files containing study data will be de-identified and password protected with access only available to study investigators. All research notes and data will be kept in a securely locked filing cabinet at The University of Queensland School of Human Movement and Nutrition Sciences. The investigators will comply with the Good Clinical Practice (GCP) guidelines adopted by the Therapeutic Goods Administration and document all adverse events through the Human Research Ethics Committee (HREC). The study investigators will permit study-related monitoring, audits, and inspections by Mater HREC of all study related documents (e.g. source documents, regulatory documents, data collection instruments, study data) and study facilities (e.g. diagnostic laboratory). This study will be conducted in full conformance with the principles of the ‘Declaration of Helsinki’ according to international standards of GCP guidelines, applicable Australian government regulations and Institutional research policies and procedures.

## Discussion

CRF is a distressing and prolonged symptom associated with prostate cancer treatment. Management of CRF is critical for improving quality of life during and after treatment for prostate cancer [4, 5, 82]. Nutrition therapy and exercise prescription have the potential to improve CRF and other prostate cancer disease- and treatment-related side effects [19–21, 25, 83–85]. This clinical trial is the first to investigate the efficacy of nutrition therapy above the healthy eating guidelines in managing prostate CRF. The lack of dietary guidelines for men with prostate cancer is a limitation in the current treatment and management of prostate cancer; thus investigating individual structured nutrition therapy provides novel insight to the efficacy of dietary modification in managing CRF and other side effects from ADT. This project will expand the exercise oncology literature by investigating the efficacy of high intensity interval training for reducing CRF and improving quality of life in men with prostate cancer. Considering both individual nutrition therapy beyond general healthy eating guidelines and high intensity interval training are yet to be investigated in men with prostate cancer, this landmark study will provide novel evidence to support the development of guidelines to optimise the management of CRF and other side effects from ADT. It is hypothesized that nutrition therapy alone, and with high intensity interval training will improve CRF and ADT-related side effects.

Dissemination of the results from this study to oncologists, urologists, prostate cancer nurses, dietitians, nutritionists, exercise physiologists and other exercise specialists will be important to ensure an evidence-based approach to the use of nutrition and exercise in the management of prostate CRF. Currently CRF is managed primarily with medications. Natural therapies such as diet and exercise provide a multi-faceted approach to managing CRF, which may in turn improve other associated side effects seen from ADT (e.g. metabolic and cardiovascular risk). With the rising incidence of prostate cancer and increasing survival rates, the primary outcome from this study will be to provide evidence to enhance clinical practice in nutrition, diet and exercise therapy to improve the lives of men suffering from the disease- and treatment-related side effects of prostate cancer.

## Abbreviations

ADT: Androgen deprivation therapy
APMHR: Age predicted maximum heart rate
CRF: Cancer related fatigue
DASS: Depression Anxiety Stress Scale
DXA: Dual energy X-ray absorptiometry
EORTC QLQ-C30: European Organization for Research and Treatment of Cancer quality of life questionnaire for people with cancer
EORTC QLQ-PR25: European Organization for Research and Treatment of Cancer prostate cancer specific quality of life questionnaire for people with cancer
FACT-F: The Functional Assessment of Cancer Therapy: Fatigue
HR_peak_: Heart rate peak
IGF: Insulin-like growth factor
IL: Interleukin
ISAK: International Society for the Advancement of Kinathropometry
ISI: Insomnia Severity Index
PACES: Physical activity enjoyment scale
RM: Repetition maximum
RPE: Rating of perceived exertion
SF-36: Short form 36 health survey
VO_2peak_: Peak volume of oxygen consumption
